# Expression of substance P, neurokinin 1 receptor, Ki-67 and pyruvate kinase M2 in hormone receptor negative breast cancer and evaluation of impact on overall survival

**DOI:** 10.1186/s12885-023-10633-8

**Published:** 2023-02-16

**Authors:** Maha S. Al-Keilani, Roba Bdeir, Rana I. Elstaty, Mohammad A. Alqudah

**Affiliations:** 1 College of Pharmacy, Department of Clinical Pharmacy, Jordan University of Science and Technology, P.O. Box 3030, 22110 Irbid, Jordan; 2 College of Nursing, Department of Allied Health Sciences, Al-Balqa Applied University, Al-Salt 19117, P.O. Box 206, Salt, Jordan; 3 College of Science and Art, Department of Biotechnology and Genetic Engineering, Jordan University of Science and Technology, P.O. Box 3030, 22110 Irbid, Jordan; 4 College of Medicine, Department of Microbiology, Pathology, and Forensic Medicine, The Hashemite University, P.O. Box 330127, 13133 Zarqa, Jordan

**Keywords:** Substance P, Neurokinin 1 receptor, Pyruvate kinase M2, Ki-67, Breast cancer, Prognosis, Biomarker, Invasive ductal carcinoma, Proliferation index

## Abstract

**Background:**

Chronic inflammation is a hallmark of cancer, and it can be stimulated by many factors. Substance P (SP), through binding to neurokinin 1 receptor (NK1R), and pyruvate kinase M2 (PKM2) play critical roles in cancer development and progression via modulating the tumor microenvironment. This study aimed to investigate the prognostic significance of SP and PKM2 in combination with NK1R and Ki-67 in hormone receptor negative (HR-ve) breast cancer.

**Methods:**

Immunohistochemical expression levels of SP, NK1R, PKM2, and Ki-67 were measured in 144 paraffin-embedded breast cancer tissues (77 h -ve and 67 h + ve). SP, NK1R, and PKM2 were scored semiquantitatively, while Ki-67 was obtained by the percentage of total number of tumor cells with nuclear staining. The optimal cutoff value for SP, NK1R, PKM2, and Ki-67 were assessed by Cutoff Finder.

**Results:**

High SP expression in HR -ve breast cancer was associated with TNM stage (p = 0.020), pT stage (p = 0.035), pN stage (p = 0.002), axillary lymph node metastasis (p = 0.003), and NK1R expression level (p = 0.010). In HR + ve breast cancer, SP expression was associated with HER2 status (p = 0.001) and PKM2 expression level (p = 0.012). Regarding PKM2 expression level, it significantly associated with HER2 status (p = 0.001) and history of DCIS (p = 0.046) in HR-ve tumors, and with HER2 status (p < 0.001) and SP expression level (p = 0.012) in HR + ve tumors. Survival analysis revealed that high SP level negatively impacted overall survival in HR-ve tumors that had low NK1R level (p = 0.021). Moreover, high SP negatively impacted overall survival in HR-ve tumors that had low Ki-67 level (p = 0.005). High PKM2 negatively impacted overall survival in HR-ve cases with low SP (p = 0.047).

**Conclusion:**

Combined expression levels of SP with NK1R or Ki-67, and PKM2 with SP could be used to predict survival in breast cancer patients with HR-ve tumors. Our findings suggest a role of SP/NK1R pathway and PKM2 in HR-ve breast cancer pathogenesis which should be further investigated to unveil the underlying molecular mechanisms.

## Background

Breast cancer is the most common cancer among women worldwide accounting for one third of newly diagnosed cancer cases in 2022 and is also ranked as the second leading cause of cancer-related mortality [1]. Immunohistochemical expression status of four biomarkers; hormone receptors [estrogen receptor (ER), progesterone receptor (PR)], human epidermal growth factor receptor (HER2), and Ki-67, are key determinants of breast cancer molecular subtyping [2]. Although accounts for about 20–30% of cases, hormone receptor negative tumors (HR-ve) are a clinical concern due to unresponsiveness to hormonal therapy and the vast heterogeneity in terms of differentiation and prognosis [3, 4]. Consequently, there is a need for the identification of new biomarkers to provide an additional clinically more relevant molecular classification of breast cancer.

Chronic inflammation is a hallmark of cancer, and it can be stimulated by many factors including inflammatory mediators such as cytokines, infectious pathogens, imbalanced immune regulation, obesity, and genetic alterations leading to overexpression of oncogenes or downregulation of tumor suppressor genes [5]. Since the discovery of the presence of leukocytes in tumor microenvironment by Rudolf Virchow in 1863, chronic inflammation has been investigated as a contributing factor to tumor initiation, development, progression, and metastasis [6]. Although the mechanism is not yet well understood, this may occur due to complex interactions mediated by production of cytokines and pro-inflammatory mediators and the subsequent activation of the associated signaling pathways [5]. Thus, better understanding the biology of the tumors and the tumor microenvironment is of outstanding benefit to identify molecular biomarkers that represent novel anticancer targets.

In breast cancer, a state of increased tissue inflammation with higher inflammatory infiltrates was observed and significantly associated with ER negative status [7, 8], while higher inflammation was associated with more aggressive tumor, poor response to endocrine therapy, and poor prognosis in ER positive breast cancer [9]. This highlights the differential effects of inflammation in breast cancer by hormone receptor status.

**Fig. 1:**
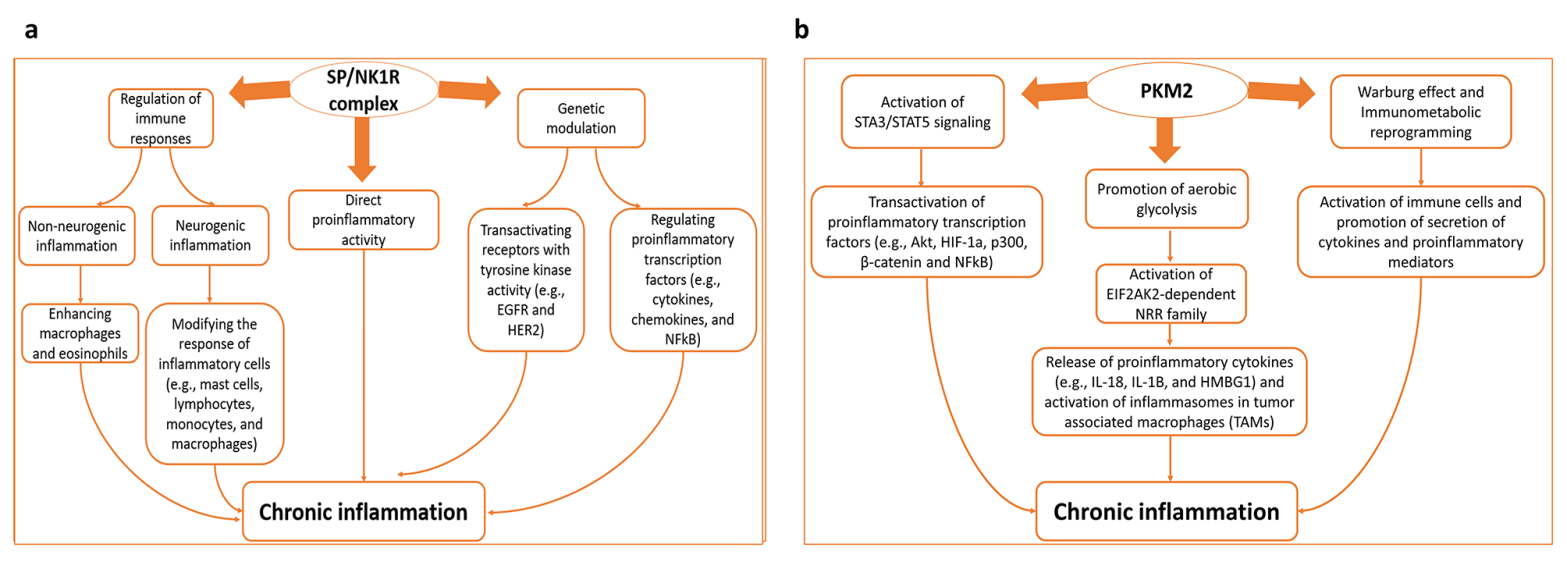
**Diagrammatic presentation of the role of SP, NK1R and PKM2 in chronic inflammation in cancer. a).** SP/NK1R complex mechanisms in chronic inflammation. SP/NK1R complex regulates immune cell function via neurogenic and nonneurogenic pathways; neurogenic inflammation occurs through the modification of inflammatory cells such as mast cells, lymphocytes, monocytes, and macrophages in tumor and peritumoral tissues. Promotion of nonneurogenic inflammation occurs through activation of macrophages and eosinophils. Additionally, SP acts as a direct proinflammatory cytokine, thus enhancing inflammation in tumor tissues. Lastly, SP/NK1R complex causes genetic modulation through transactivation of receptors with tyrosine kinase activity such as EGFR and HER2, and regulation of proinflammatory transcription factors such as NFkB, cytokines, and chemokines. **b).** PKM2 mechanisms in chronic inflammation. PKM2 activates proinflammatory transcription factors such as Akt, HIF-1a, p300, β-catenin, and NFkB. Moreover, it promotes aerobic glycolysis which results in activation of E1F2AK2-dependent NRR family and the release of proinflammatory cytokines such as IL-18, IL-1B, and HMBG1, and the activation of inflammasomes in TMAs. Lastly, PKM2-mediated Warburg effect and immunometabolic reprogramming causes activation of immune cells and promotes the secretion of cytokines and proinflammatory mediators

Substance P (SP), an undecapeptide, is a neuropeptide of the pro-inflammatory tachykinin family. SP plays an integral role in peripheral inflammation via specific binding to a transmembrane G-protein coupled receptor; neurokinin 1 receptor (NK1R)present on epithelial cells, and immune and inflammatory cells such as macrophages and T-lymphocytes [10]. In terms of cancer, SP/NK1R receptor complex was discovered to play an important role in maintaining a favorable tumor microenvironment which was associated with induced mitogenesis, angiogenesis, cancer cell migration, and metastasis [11]. In this respect, an altered SP/NK1R signaling in cancer was evident in different types of tumors one of them is breast cancer [12–17], and both proteins were overexpressed in several cancers including breast cancer [10, 17–24]. A diagrammatic presentation for the roles of SP/NK1R complex in chronic inflammation is shown in Fig. 1a.

Pyruvate kinase M2 (PKM2), is the final rate-limiting enzyme in the aerobic glycolysis pathway where it catalyzes the formation of pyruvate and ATP from phosphoenolpyruvate and ADP. PKM2 was shown to play an important role in Warburg effect in tumor and immune cells, a key metabolic process that favors tumor cell proliferation [25]. Moreover, it has been implicated in immunometabolic reprogramming resulting in excessive inflammation in cancer, and was upregulated in various types of tumors [26–32].

A diagrammatic presentation for the roles of PKM2 in chronic inflammation is shown in Fig. 1b.

Few studies investigated the prognostic potential of SP, NK1R, and PKM2 in breast cancer [20, 21]. However, these studies had small sample size and none of them examined the effect of combined expression of these target markers in different molecular subtypes of breast cancer. Our previous study suggested that NK1R is a promising prognostic marker in breast cancer, where NK1R expression negatively impacted overall survival in patients with grade II breast cancer [24]. In another study, we also revealed that SP was overexpressed in breast cancer [22]. The aim of the present study was to examine the prognostic value of combined expression of SP with NK1R, PKM2, and Ki67 and their association with various prognostic factors in hormone receptor negative breast cancer.

## Materials and methods

### Patients

The present study included a total of 144 cases of breast cancer specimens that were examined at the pathology department of King Abdulla University Hospital (Irbid, Jordan) from 2007 to 2019. Patients were excluded if they had received chemotherapy or radiotherapy prior to surgery, or in case of missing clinicopathological data or no paraffin-embedded blocks containing tumor tissue. The clinicopathological data obtained from the medical charts of the patients included patient’s age, tumor grade, TNM stage, pT stage, pN stage, site of metastasis, lymphovascular invasion, axillary lymph node metastasis, tumor size, history of ductal carcinoma in situ (DCIS), HR status, HER2 status, and family history of breast cancer.

### Tissue microarray (TMA) construction and immunohistochemical staining (IHC)

TMA and IHC were performed as described previously [22, 24]. Briefly, three cores from each case were taken from regions of interest and used to construct the TMA paraffin-embedded blocks using TMA Master II instrument (3DHISTECH Ltd., Budapest, Hungary). TMA tissue blocks were sectioned at 4-µm thickness and collected on Superfrost plus glass slides for processing by IHC using the BenchMark ULTRA system (Roche Diagnostics, Risch-Rotkreuz, Switzerland). IHC was performed on 4-µm sections of the breast carcinoma, using a BenchMark ULTRA system (Roche Diagnostics, Risch-Rotkreuz, Switzerland), following a standard protocol, as per the manufacturer’s recommendations. Primary antibodies for the target proteins SP (1:50, Abcam, Cat# ab10353, RRID:AB_297089), NK1R (1:50, ab219600; RRID is not available), PKM2 (1:100, Cat# ab38237, RRID:AB_777576), and Ki-67 (clone 30–9, prediluted, #790–4286, RRID:AB_2631262) were used in this study.

### Immunostaining evaluation and interpretation

Immunopositivity was assessed blindly by three independent pathologists without prior knowledge of the clinical outcome. Assessment was based on two parameters for SP, NK1R, and PKM2: the staining intensity score (0 = negative, 1 = weak, 2 = moderate, and 3 = strong) and the percentage of immunopositive cells (0-100) to create a proportion score (0, 0% reacting cells; 1, 1-25% reacting cells; 2, 26-50% reacting cells; 3, 51-75% reacting cells; and 4, > 75% reacting cells [33]. The two scores were added to yield a final score [Total score (TS) = 0–7].

The cut-off value for each marker’s score was determined using Cutoff Finder [34]. Accordingly, two groups were stratified based on that: low expression (TS ≤ cut-off value), and high expression group (TS > cut-off value).

Ki-67 immunostaining was evaluated based on the average percentage of positively stained cells considering positive nuclear staining only regardless of immunostaining intensity.

**Fig. 2:**
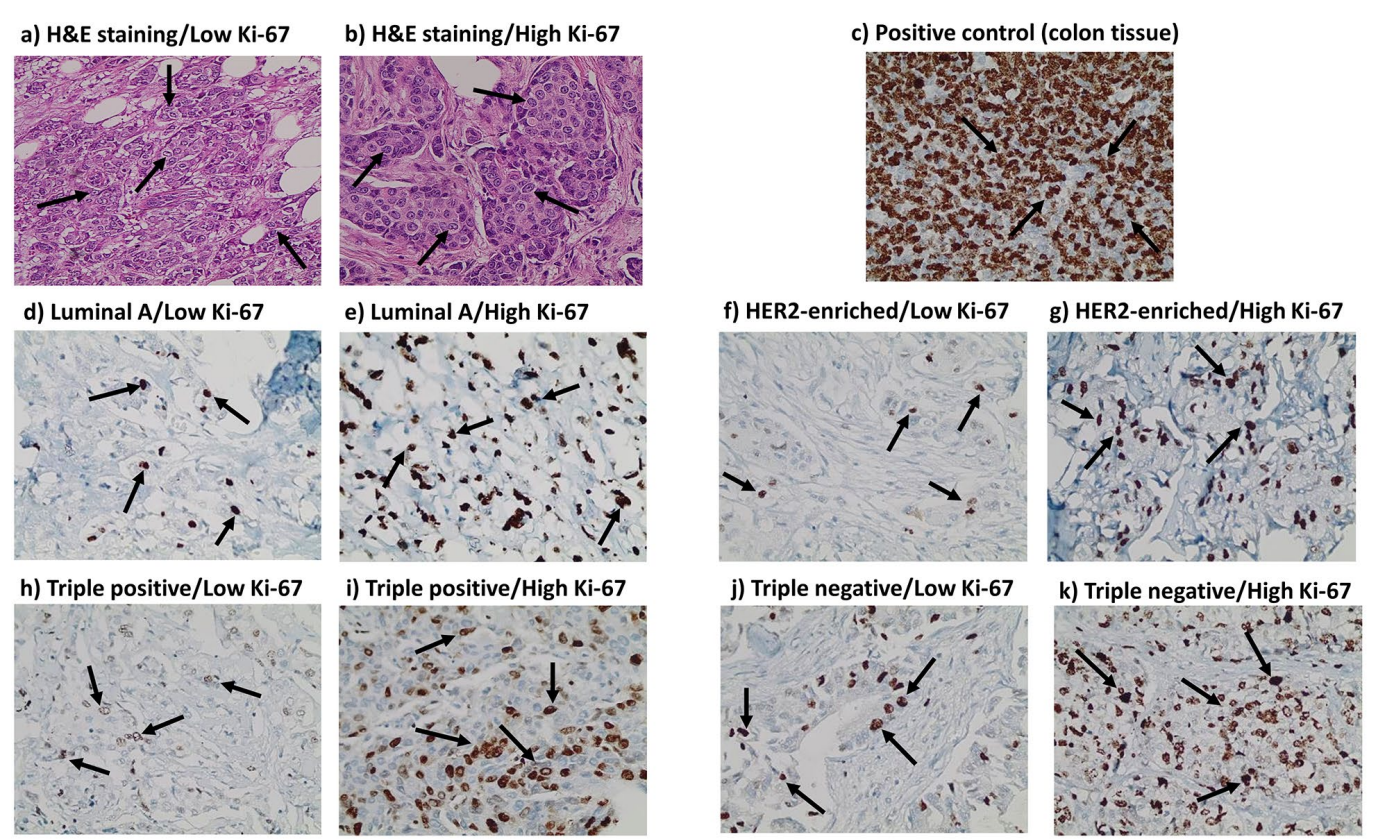
** H&E and immunohistochemistry (IHC) staining for Ki-67 expression in breast cancer tissues and positive control**. **a).** H&E staining of low Ki-67 expression in breast cancer tissue. **b).** H&E staining of high Ki-67 expression in breast cancer tissue. **c-k).** IHC staining of Ki-67 manifested in the nucleus in: **c).** Positive control tissue (colon tissue). **d).** Luminal A breast cancer subtype (low expression level ≤ 22%). **e).** Luminal A breast cancer subtype (high expression level > 22%). **f).** HER2-enriched breast cancer subtype (low expression level ≤ 22%). **g).** HER2-enriched breast cancer subtype (high expression level > 22%). **h).** Triple positive breast cancer subtype (low expression level ≤ 22%). **i).** Triple positive breast cancer subtype (high expression level > 22%). **j).** Triple negative breast cancer subtype (low expression level ≤ 22%). **k).** Triple negative breast cancer subtype (high expression level > 22%). Arrows indicate the positively stained cells. Scale bar: 400 × (40X objective lens x 10X ocular lens = 20 μm).

**Fig. 3:**
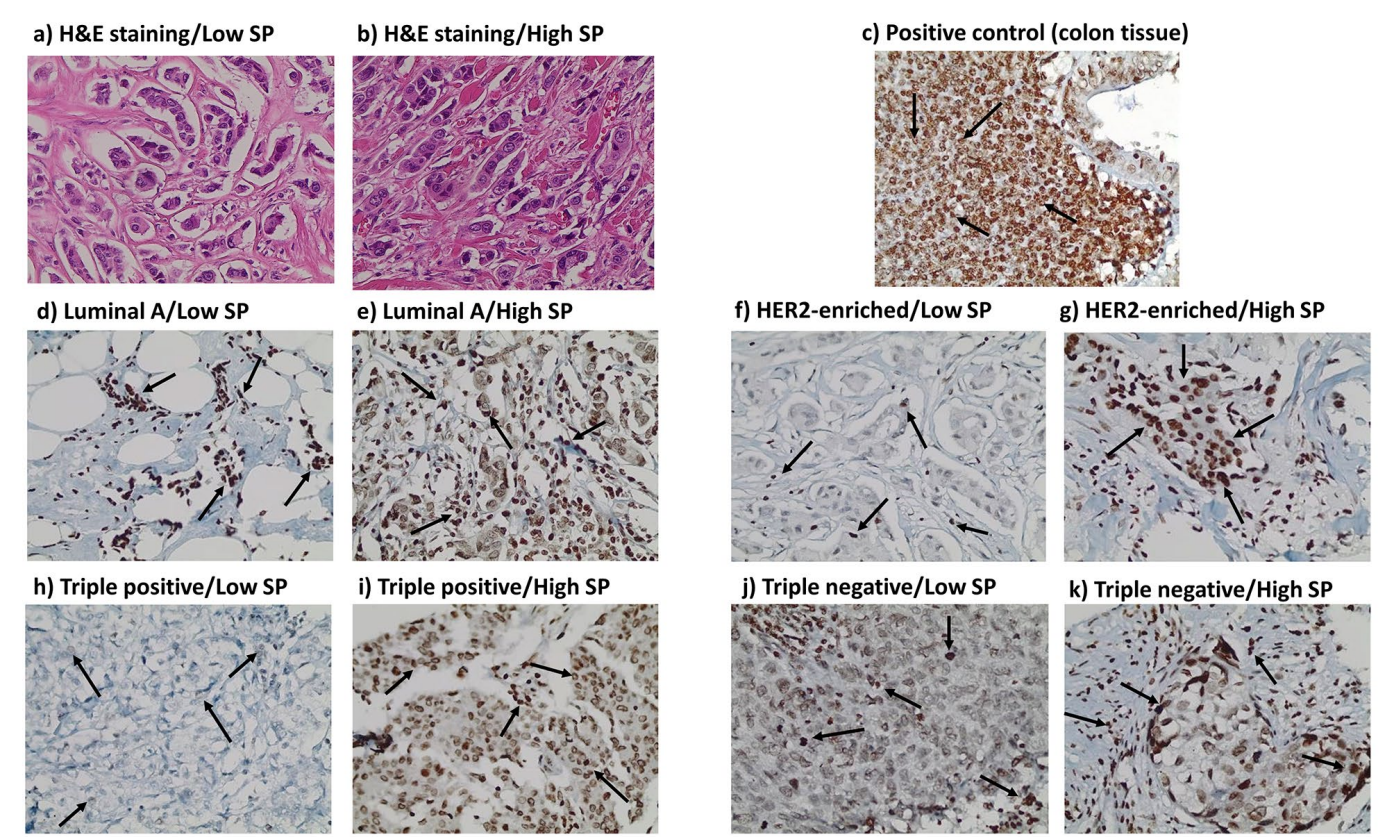
** H&E and immunohistochemistry (IHC) staining for substance p (SP) expression in breast cancer tissues and positive control**. **a).** H&E staining of low SP expression in breast cancer tissue. **b).** H&E staining of high SP expression in breast cancer tissue. **c-k).** IHC staining of SP manifested mainly in the nucleus in: **c).** Positive control tissue (colon tissue). **d).** Luminal A breast cancer subtype (low expression level ≤ 5). **e).** Luminal A breast cancer subtype (high expression level > 5). **f).** HER2-enriched breast cancer subtype (low expression level ≤ 5). **g).** HER2-enriched breast cancer subtype (high expression level > 5). **h).** Triple positive breast cancer subtype (low expression level ≤ 5). **i).** Triple positive breast cancer subtype (high expression level > 5). **j).** Triple negative breast cancer subtype (low expression level ≤ 5). **k).** Triple negative breast cancer subtype (high expression level > 5). Arrows indicate the positively stained cells. Scale bar: 400 × (40X objective lens x 10X ocular lens = 20 μm)

**Fig. 4:**
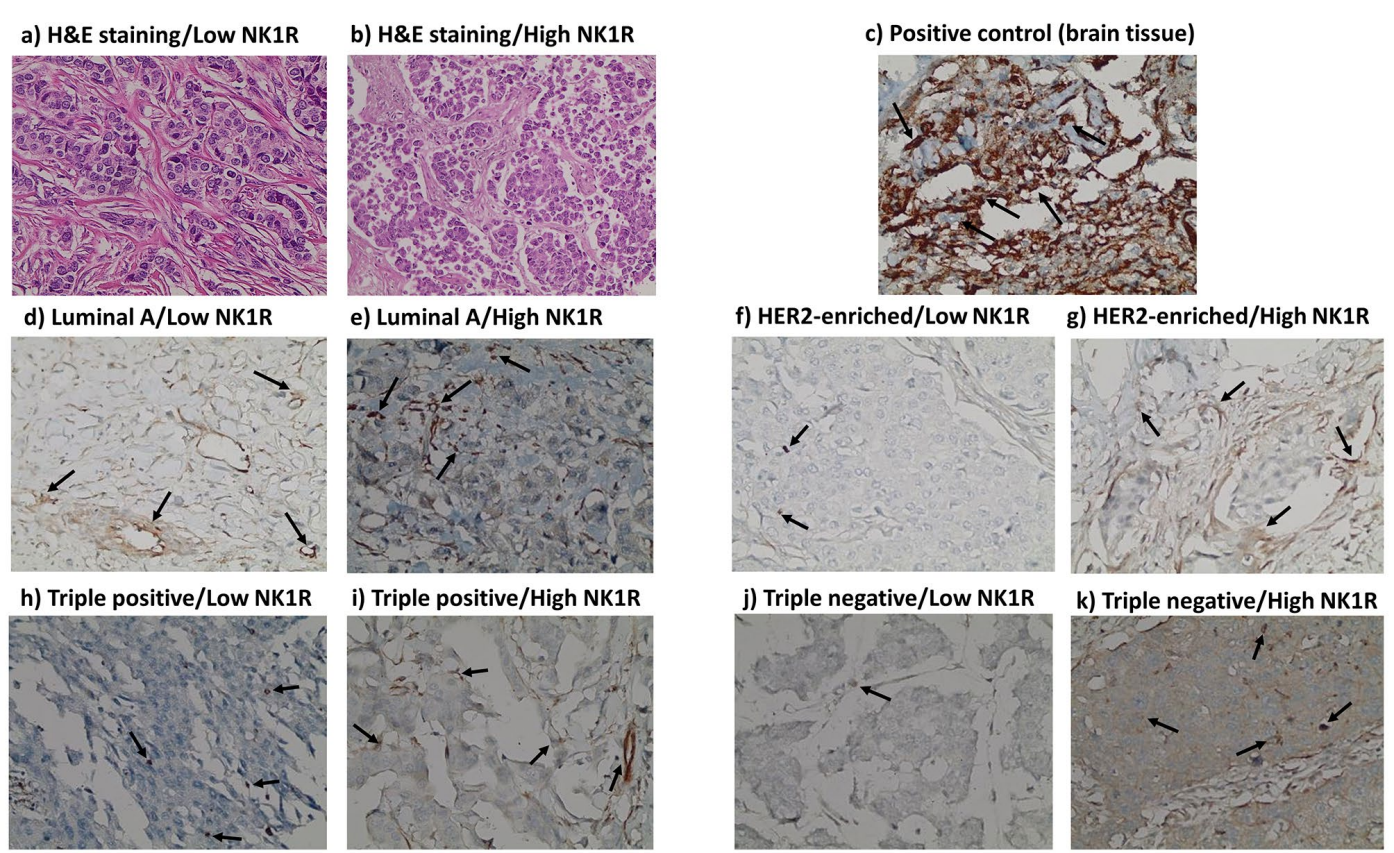
** H&E and immunohistochemistry (IHC) staining for neurokinin 1 receptor (NK1R) expression in breast cancer tissues and positive control**. **a).** H&E staining of low NK1R expression in breast cancer tissue. **b).** H&E staining of high NK1R expression in breast cancer tissue. **c-k).** IHC staining of NK1R manifested mainly in nucleus and cytoplasm in: **c).** Positive control tissue (brain tissue). **d).** Luminal A breast cancer subtype (low expression level ≤ 1). **e).** Luminal A breast cancer subtype (high expression level > 1). **f).** HER2-enriched breast cancer subtype (low expression level ≤ 1). **g).** HER2-enriched breast cancer subtype (high expression level > 1). **h).** Triple positive breast cancer subtype (low expression level ≤ 1). **i).** Triple positive breast cancer subtype (high expression level > 1). **j).** Triple negative breast cancer subtype (low expression level ≤ 1). **k).** Triple negative breast cancer subtype (high expression level > 1). Arrows indicate the positively stained cells. Scale bar: 400 × (40X objective lens x 10X ocular lens = 20 μm)

**Fig. 5:**
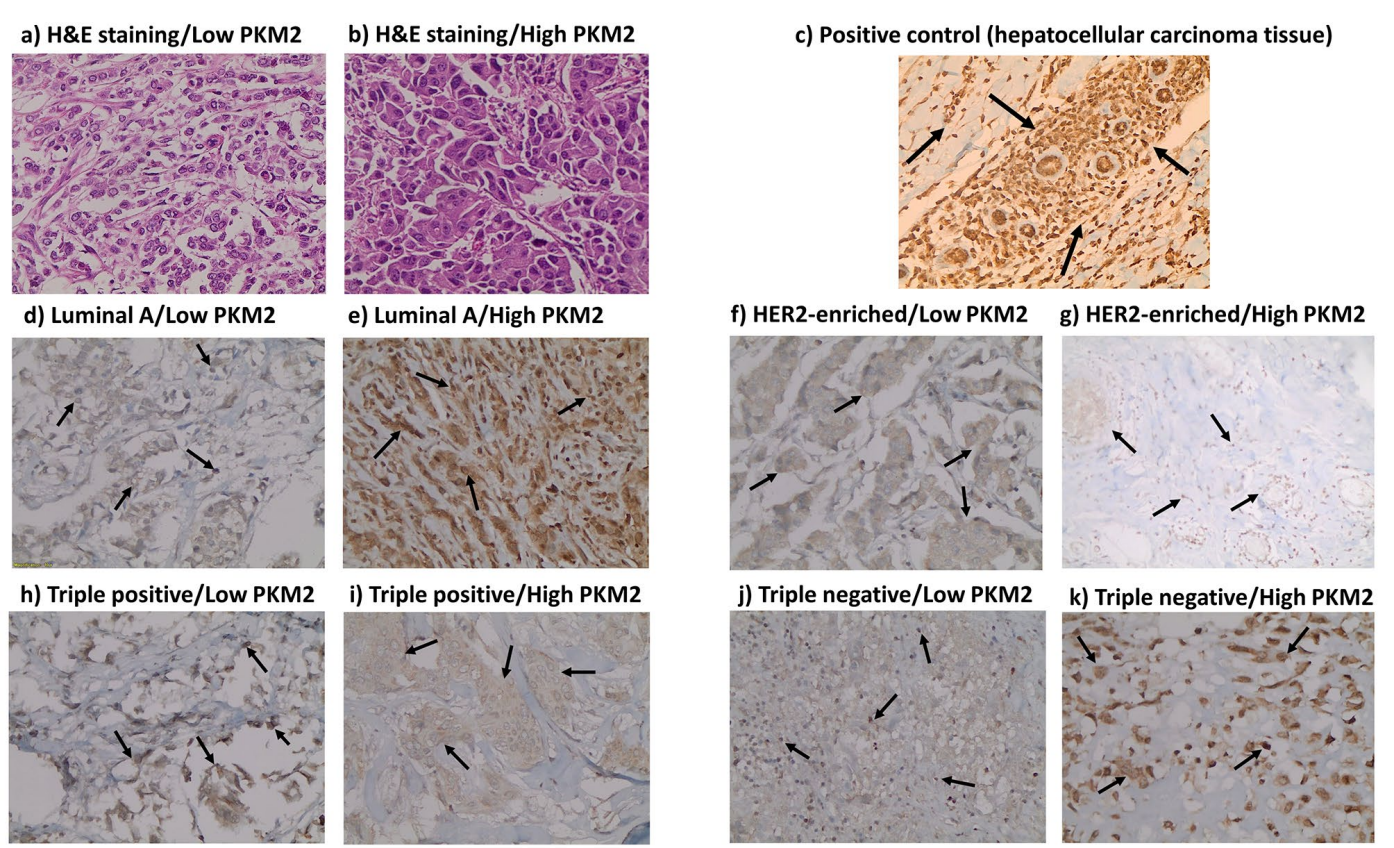
** H&E and immunohistochemistry (IHC) staining for pyruvate kinase M2 (PKM2) expression in breast cancer tissues and positive control**. **a).** H&E staining of low PKM2 expression in breast cancer tissue. **b).** H&E staining of high PKM2 expression in breast cancer tissue. **c-k).** IHC staining of PKM2 manifested mainly in the nucleus in: **c).** Positive control tissue (colon tissue). **d).** Luminal A breast cancer subtype (low expression level ≤ 4). **e).** Luminal A breast cancer subtype (high expression level > 4). **f).** HER2-enriched breast cancer subtype (low expression level ≤ 4). **g).** HER2-enriched breast cancer subtype (high expression level > 4). **h).** Triple positive breast cancer subtype (low expression level ≤ 4). **i).** Triple positive breast cancer subtype (high expression level > 4). **j).** Triple negative breast cancer subtype (low expression level ≤ 4). **k).** Triple negative breast cancer subtype (high expression level > 4). Arrows indicate the positively stained cells. Scale bar: 400 × (40X objective lens x 10X ocular lens = 20 μm)

SP was primarily expressed in the nucleus, NK1R in the nucleus and cytoplasm, while PKM2 was expressed mainly in the nucleus of tumor cells. Representative IHC pictures for Ki-67, SP, NK1R, and PKM2 are shown in Figs. 2, 3, 4 and 5.

### Statistical analysis

Statistical package for social sciences (SPSS 23) was used for data entry and analysis. Descriptive statistics were done. The associations between categorical variables were evaluated by Chi-square test or Fisher’s Exact test as appropriate. Student t-test was applied to compare difference in means among groups. Continuous variables were reported as mean ± SD, while categorical variables were reported as numbers and percentages. Overall survival was defined as the period from time of diagnosis to death from any cause or the last follow up. Kaplan Meier analysis was used to estimate the overall survival, and Log rank test was used to compare survival curves. A probability p-value ≤ 0.05 was considered statistically significant.

## Results

### Clinicopathological features of breast cancer patients

All patients were females whose ages ranged from 28 to 82 years, with mean age of 51.61 years. All cases were invasive ductal carcinoma. About two third of cases were stages III and IV (67.4%). Grade III tumors were predominant (70.8%). The positivity of HR (ER/PR) and HER2 were 46.5% and 54.2%, respectively. Forty-two cases (29.2%) were HER2-enriched (ER-/PR-, HER2+), 31 cases (21.5%) were luminal A (ER+/PR+, HER2-), 36 cases (25.0%) were triple positive, and 35 (24.3%) were triple negative. The clinicopathological characteristics of breast cancer patients are presented in Table 1.

**Table 1:** Clinicopathological characteristics of breast cancer patients (n = 144)

Variable	Total (n%)
**Age (Years)**	
Mean ± SD	51.61 ± 11.59
Range	28–82
**Breast cancer molecular subtype**	
HER2-enriched	42 (29.2)
Luminal A	31 (21.5)
Triple positive	36 (25.0)
Triple negative (TNBC)	35 (24.3)
**Grade**	
I	7 (4.9)
II	35 (24.3)
III	102 (70.8)
**Tumor volume (cm** ^**3**^ **)**	
Mean ± SD	41.00 ± 70.88
Range	0.11-571.77
**TNM Stage**	
I	7 (4.9)
II	37 (25.7)
III	48 (33.3)
IV	49 (34.0)
Undetermined	3 (2.1)
**pT**	
T1	11 (7.6)
T2	74 (51.4)
T3	44 (30.6)
T4	15 (10.4)
**pT**	
T1/T2	85 (59.0)
T3/T4	59 (41.0)
**pN**	
N0	40 (27.8)
N1	31 (21.5)
N2	29 (20.1)
N3	39 (27.1)
Undetermined	5 (3.5)
**M**	
M0	92 (63.9)
M1	49 (34.0)
Undetermined	3 (2.1)
**Axillary lymph node metastasis**	
Negative	41 (28.5)
Positive	101 (70.1)
Undetermined	2 (1.4)
**Lymphatic/vascular invasion**	
Negative	47 (32.6)
Positive	91 (63.2)
Undetermined	6 (4.2)
**HR status**	
Negative	77 (53.5)
Positive	67 (46.5)
**HER-2 status**	
Negative	66 (45.8)
Positive	78 (54.2)
**DCIS**	
Absent	32 (22.2)
Present	109 (75.7)
Undetermined	3 (2.1)
**Family history**	
No	90 (62.5)
Yes	25 (17.4)
Undetermined	29 (20.1)
**SP**	
Low (TS ≤ 5)	85 (59.0)
High (TS > 5)	59 (41.0)
**NK1R**	
Low (TS ≤ 1)	91 (63.2)
High (TS > 1)	53 (36.8)
**Ki-67**	
Low (≤ 22%)	83 (57.6)
High (> 22%)	61 (42.2)
**PKM2**	
Low (TS ≤ 4)	78 (54.2)
High (TS > 4)	55 (38.2)
Undetermined	11 (7.6)

SD: standard deviation, HR: hormone receptor, HER2: human epidermal growth factor receptor 2, DCIS: ductal carcinoma in situ, SP: substance P, TS: total score, NK1R: neurokinin 1 receptor, PKM2: pyruvate kinase M2

Immunohistochemical analysis of breast cancer tissues showed that 41% (59/144) expressed high SP (TS > 5), 36.8% (53/144) expressed high NK1R (TS > 1), 42.2% (61/144) expressed high Ki-67 (expression > 22%), and 38.2% (55/133) expressed high PKM2 (TS > 4).

### Association between SP and PKM2 expression with clinicopathological parameters in HR-ve versus HR + ve breast cancer

As shown in Table 2, in HR-ve tumors there were significant associations between SP expression level and TNM stage (p = 0.020), pT stage (p = 0.035), pN stage (p = 0.002), axillary lymph node metastasis (p = 0.003), and NK1R expression level (p = 0.010). Whereas in HR + ve tumors, there was a significant association with HER2 status (p = 0.001) and PKM2 expression level (p = 0.012).

**Table 2:** Relationship between SP expression and clinicopathological features of breast cancer patients with HR-ve versus HR + ve tumors

Parameters	HR-ve (n = 77)			HR + ve (n = 67)		
	Low SP	High SP	p-value	Low SP	High SP	p-value
**Age (Years)**	51.77 ± 12.97	49.97 ± 10.76	0.529	52.26 ± 12.50	52.21 ± 8.95	0.984
**Tumor grade** I II III	0 (0) 7 (14.9) 40 (85.1)	0 (0) 4 (13.3) 26 (86.7)	1.000	4 (10.5) 13 (34.2) 21 (55.3)	3 (10.3) 11 (37.9) 15 (51.7)	0.936
**Tumor volume (cm** ^**3**^ **)**	62.28 ± 105.25	34.85 ± 47.08	0.184	33.53 ± 47.53	22.67 ± 30.91	0.289
**TNM Stage** I II III IV	3 (6.5) 16 (34.8) 16 (34.8) 11 (23.9)	1 (3.4) 3 (10.3) 12 (41.4) 13 (44.8)	0.056	1 (2.6) 9 (23.7) 11 (28.9) 17 (44.7)	2 (7.1) 9 (32.1) 9 (32.1) 8 (28.6)	0.527
**TNM stage** I/II III/IV	19 (41.3) 27 (58.7)	4 (13.8) 25 (86.2)	**0.020**	10 (26.3) 28 (73.7)	11 (39.3) 17 (60.7)	0.295
**T stage** T1 T2 T3 T4	3 (6.4) 28 (59.6) 12 (25.5) 4 (8.5)	3 (10.0) 9 (30.0) 13 (43.3) 5 (16.7)	0.081	3 (7.9) 19 (50.0) 11 (28.9) 5 (13.2)	2 (6.9) 18 (62.1) 8 (27.6) 1 (3.4)	0.569
**pT** T1/T2 T3/T4	31 (72.1) 16 (47.1)	12 (27.9) 18 (52.9)	**0.035**	22 (52.4) 16 (64.0)	20 (47.6) 9 (36.0)	0.447
** N stage** N0 N1 N2 N3	18 (39.1) 7 (15.2) 12 (26.1) 9 (19.6)	2 (7.1) 8 (28.6) 4 (14.3) 14 (50.0)	**0.002**	12 (32.4) 6 (16.2) 8 (21.6) 11 (29.7)	8 (28.6) 10 (35.7) 5 (17.9) 5 (17.9)	0.332
**M stage** M0 M1	35 (76.1) 11 (23.9)	16 (55.2) 13 (44.8)	0.077	21 (55.3) 17 (44.7)	20 (71.4) 8 (28.6)	0.208
**Axillary lymph nodes metastasis** Negative Positive	18 (39.1) 28 (60.9)	2 (6.9) 27 (93.1)	**0.003**	12 (31.6) 26 (68.4)	9 (31.0) 20 (69.0)	1.000
**Lymphatic/vascular invasion** Negative Positive	18 (41.9) 25 (58.1)	6 (20.7) 23 (79.3)	0.077	12 (31.6) 26 (28.4)	11 (39.3) 17 (60.7)	0.604
**HER2 status** Negative Positive	24 (51.1) 23 (48.9)	11 (36.7) 19 (63.3)	0.248	11 (28.9) 27 (71.1)	20 (69.0) 9 (31.0)	**0.001**
**DCIS** Absent Present	16 (36.4) 28 (63.6)	6 (20.0) 24 (80.0)	0.195	7 (18.4) 31 (81.6)	3 (10.3) 26 (89.7)	0.495
**Family History** No Yes	28 (80.0) 7 (20.0)	11 (61.1) 7 (38.9)	0.191	28 (84.8) 5 (15.2)	23 (79.3) 6 (20.7)	0.741
**Ki-67** Low High	21 (44.7) 26 (55.3)	14 (46.7) 16 (53.3)	1.000	28 (73.7) 10 (26.3)	20 (69.0) 9 (31.0)	0.786
**NK1R** Low High	33 (70.2) 14 (29.8)	12 (40.0) 18 (60.0)	**0.010**	24 (63.2) 14 (68.8)	22 (75.9) 7 (24.1)	0.300
**PKM2** Low High	24 (58.5) 17 (41.5)	18 (69.2) 8 (30.8)	0.444	26 (68.4) 12 (31.6)	10 (35.7) 18 (64.3)	**0.012**

HR: hormone receptor, HER2: human epidermal growth factor receptor 2, DCIS: ductal carcinoma in situ, SP: substance P, NK1R: neurokinin 1 receptor, PKM2: pyruvate kinase M2

As shown in Table 3, there were only significant associations between PKM2 and HER2 status (p = 0.001) and DCIS (p = 0.046) in HR-ve tumors, and with HER2 status (p < 0.001) and SP expression level (p = 0.012) in HR + ve tumors.

**Table 3:** Relationship between PKM2 expression and clinicopathological features of breast cancer patients with HR-ve versus HR + ve tumors

Parameters	HR -ve			HR + ve		
	Low PKM2	High PKM2	p-value	Low PKM2	High PKM2	p-value
**Age (Years)**	51.88 ± 12.82	48.96 ± 11.78	0.929	53.08 ± 12.27	51.53 ± 9.54	0.575
**Tumor grade** I II III	0 (0.0) 7 (16.7) 35 (83.3)	0 (0.0) 2 (8.0) 23 (92.0)	0.466	4 (11.1) 10 (27.8) 22 (61.1)	3 (10.0) 14 (46.7) 13 (43.3)	0.270
**Tumor volume (cm** ^**3**^ **)**	47.87 ± 67.56	66.64 ± 125.41	0.428	24.97 ± 34.89	34.28 ± 48.29	0.368
**TNM Stage** I II III IV	1 (2.5) 14 (35.0) 14 (35.0) 11 (27.5)	2 (8.0) 4 (16.0) 10 (40.0) 9 (36.0)	0.297	3 (8.3) 10 (27.8) 11 (30.6) 12 (33.3)	0 (0.0) 8 (27.6) 9 (31.0) 12 (41.4)	0.532
**TNM** I/II III/IV	15 (37.5) 25 (62.5)	6 (24.0) 19 (76.0)	0.290	13 (36.1) 23 (63.9)	8 (27.6) 21 (72.4)	0.595
**T stage** T1 T2 T3 T4	2 (4.8) 22 (52.4) 13 (31.0) 5 (11.9)	3 (12.0) 11 (44.0) 9 (36.0) 2 (8.0)	0.640	5 (13.9) 19 (52.8) 9 (25.0) 3 (8.3)	0 (0.0) 18 (60.0) 10 (33.3) 2 (6.7)	0.197
**pT** T1/T2 T3/T4	24 (57.1) 18 (42.9)	14 (56.0) 11 (44.0)	1.000	24 (66.7) 12 (33.3)	18 (60.0) 12 (40.0)	0.615
**pN stage** N0 N1 N2 N3	11 (28.2) 13 (33.3) 6 (15.4) 9 (23.1)	7 (28.0) 2 (8.0) 6 (24.0) 10 (40.0)	0.093	12 (34.3) 6 (17.1) 7 (20.0) 10 (28.6)	8 (27.6) 9 (31.0) 6 (20.7) 6 (20.7)	0.602
**M stage** M0 M1	29 (72.5) 11 (27.5)	16 (64.0) 9 (36.0)	0.583	24 (66.7) 12 (33.3)	17 (58.6) 12 (41.4)	0.607
**Axillary lymph nodes metastasis** Negative Positive	11 (27.5) 29 (72.5)	7 (28.0) 18 (72.0)	1.000	12 (33.3) 24 (66.7)	9 (30.0) 21 (70.0)	0.797
**Lymphatic/vascular invasion** Negative Positive	15 (38.5) 24 (61.5)	6 (26.1) 17 (73.9)	0.409	16 (45.7) 19 (54.3)	7 (23.3) 23 (76.7)	0.073
**HER2 status** Negative Positive	11 (26.2) 31 (73.2)	17 (68.0) 8 (32.0)	**0.001**	7 (19.4) 29 (80.6)	24 (80.0) 6 (20.0)	**< 0.001**
**DCIS** Absent Present	8 (19.0) 34 (81.0)	10 (43.5) 13 (56.5)	**0.046**	7 (19.4) 29 (80.6)	3 (10.0) 27 (90.0)	0.327
**Family History** No Yes	17 (70.8) 7 (29.2)	15 (68.2) 7 (31.8)	1.000	25 (78.1) 7 (21.9)	26 (89.7) 3 (10.3)	0.307
**Ki-67** Low High	19 (45.2) 23 (54.8)	11 (44.0) 14 (56.0)	1.000	24 (66.7) 12 (33.3)	24 (80.0) 6 (20.0)	0.275
**SP** Low High	24 (57.1) 18 (42.9)	17 (68.0) 8 (32.0)	0.444	26 (72.2) 10 (27.8)	12 (40.0) 18 (60.0)	**0.012**
**NK1R** Low High	26 (61.9) 16 (38.1)	12 (48.0) 13 (52.0)	0.314	24 (66.7) 12 (33.3)	21 (70.0) 9 (30.0)	0.797

HR: hormone receptor, HER2: human epidermal growth factor receptor 2, DCIS: ductal carcinoma in situ, SP: substance P, NK1R: neurokinin 1 receptor, PKM2: pyruvate kinase M2

### Prognostic analysis of SP and PKM2 expression

**Fig. 6:**
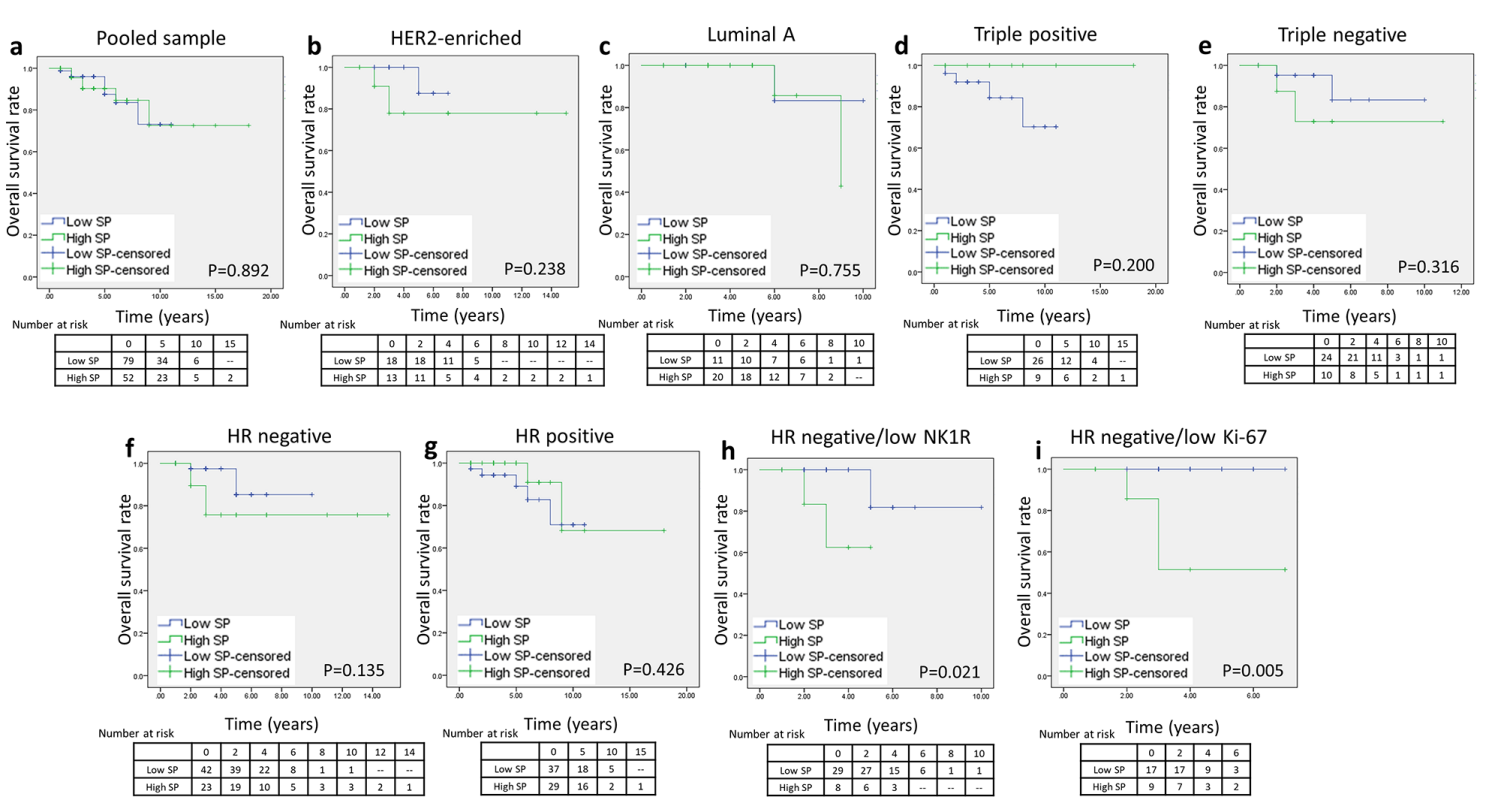
**Kaplan–Meier survival curves of overall survival for SP expression in patients with breast cancer (log-rank test). a).** Overall survival based on SP expression in pooled samples. **b).** Overall survival based on SP expression in HER2-enriched cases. **c).** Overall survival based on SP expression in luminal A cases. **d).** Overall survival based on SP expression in triple positive cases. **e).** Overall survival based on SP expression in triple negative cases. **f).** Overall survival based on SP expression in HR-ve cases. **g).** Overall survival based on SP expression in HR + ve cases. **h).** Overall survival based on SP expression HR-ve cases with low NK1R expression level. **i).** Overall survival based on SP expression in HR-ve cases with low Ki-67 index Significant differences were calculated using the log-rank test. p < 0.05 is statistically significant Abbreviations: SP: substance P; HER2: human epidermal growth factor receptor type 2; HR-ve: hormone receptor negative; HR + ve: hormone receptor positive; NK1R: neurokinin 1 receptor.

Kaplan-Meier survival analysis was performed to explore the impact of SP and PKM2 expression on overall survival. There was no significant difference between low and high SP expression when all subtypes were pooled together (Fig. 6a), as well as when each molecular subtype was independently analyzed (Fig. 6b and e) or when cases were analyzed by HR status (Fig. 6f g). However, since SP expression positively associated with NK1R expression level in HR-ve breast cancer, we further investigated its impact on overall survival where cases were grouped by HR status and NK1R expression level. As shown in Fig. 6h, there was a negative impact of SP expression level on overall survival in HR-ve cases with low NK1R expression level (p = 0.021). Since Ki-67 is an important potential prognostic marker in breast cancer, impact of SP on overall survival where cases grouped by Ki-67 expression level and HR status was also investigated. There was a negative impact of high SP expression on overall survival in HR-ve cases with low Ki-67 index (Fig. 6i, p = 0.005).

**Fig. 7:**
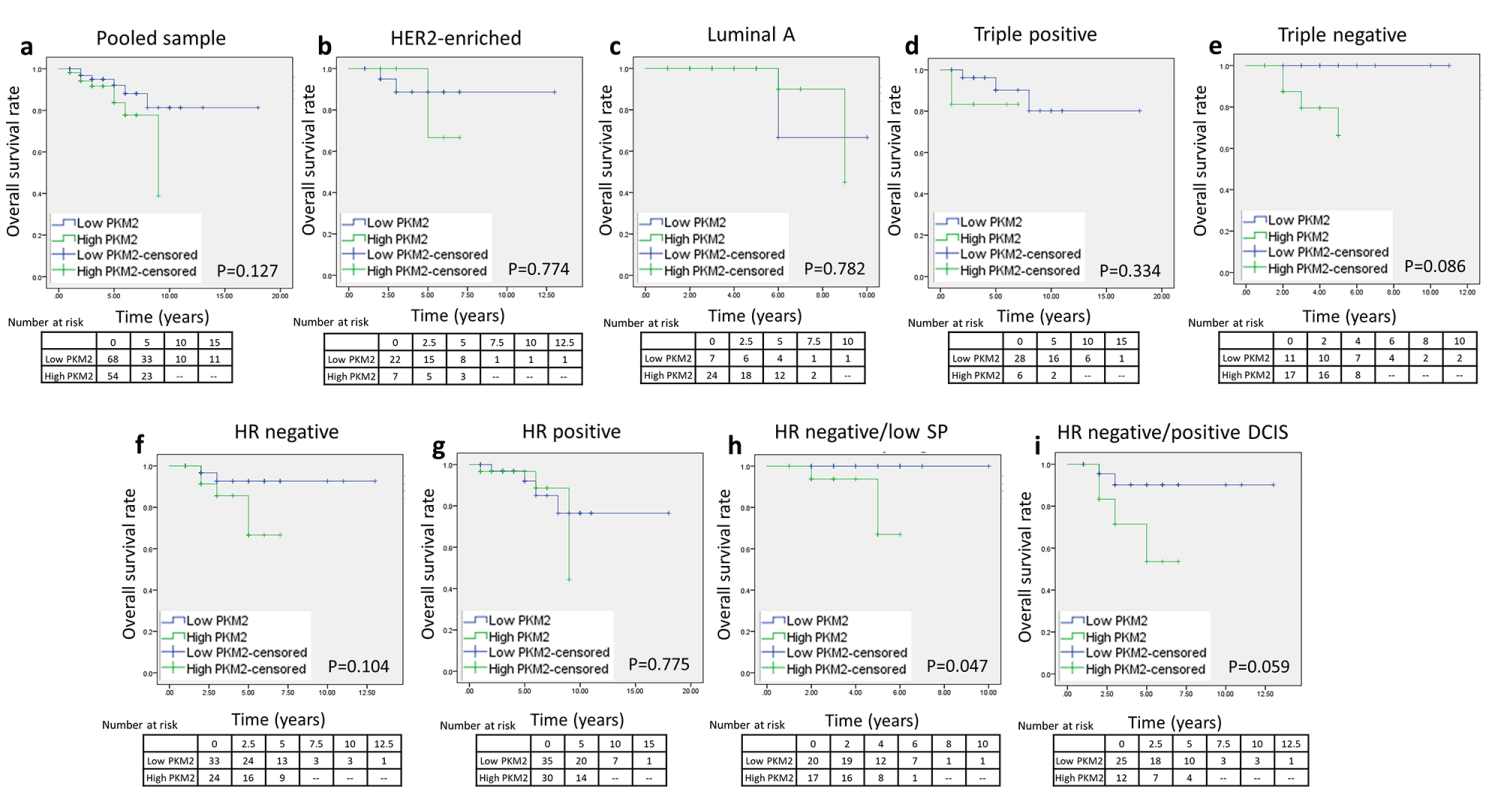
**Kaplan–Meier survival curves of overall survival for PKM2 expression in patients with breast cancer. a).** Overall survival based on PKM2 expression in pooled samples. **b).** Overall survival based on PKM2 expression in HER2-enriched cases. **c).** Overall survival based on PKM2 expression in luminal A cases. **d).** Overall survival based on PKM2 expression in triple positive cases. **e).** Overall survival based on PKM2 expression in triple negative cases. **f).** Overall survival based on PKM2 expression in HR-ve cases. **g).** Overall survival based on PKM2 expression in HR + ve cases. **h).** Overall survival based on PKM2 expression HR-ve cases with low SP expression level. **i).** Overall survival based on PKM2 expression in HR-ve cases with positive DCIS history. Significant differences were calculated using the log-rank test. p < 0.05 is statistically significant Abbreviations: PKM2: pyruvate kinase M2; HER2: human epidermal growth factor receptor type 2; HR-ve: hormone receptor negative; HR + ve: hormone receptor positive; SP: substance P; DCIS: ductal carcinoma in situ.

As revealed in Fig. 7a, we found no statistically significant difference in overall survival with low versus high PKM2 expression level when all cases were pooled together (p = 0.127), or in the different molecular subtypes (Fig. 7b and e), neither when grouped by HR status (Fig. 7f, p = 0.104 and Fig. 7g, p = 0.775). When cases were grouped by HR status and SP expression level, high PKM2 negatively impacted overall survival in HR-ve cases with low SP (Fig. 7h, p = 0.047).

It is worth to note that high PKM2 expression negatively impacted overall survival in HR-ve tumors with positive DCIS but did not reach statistical significance (Fig. 7i, p = 0.059).

## Discussion

Through activation of NK1R, the SP/NK1R system regulates the proliferation, migration, and metastasis of breast cancer cells [35]. Our previous studies revealed that SP was overexpressed in most of the analyzed tissues and was associated with prognostic factors in the breast cancer patients [22], while NK1R expression negatively impacted overall survival in patients with grade II breast cancer [24]. In this study, we investigated the prognostic potential of the SP/NK1R system in hormone receptor negative breast cancer, and its relationship with PKM2 by evaluating their expression in 144 breast cancer cases. We also tested their potential association with clinicopathological parameters as well as with the proliferative marker, Ki-67, and their impact on overall survival rate. Thus, trying to establish the link between metabolic changes occurring in cancer tissues and the role of inflammation and examining the effect of combined expression of these target markers in different molecular subtypes of breast cancer. As expected, a noteworthy percent, ranging 37-40%, of the cases had high expression of the three biomarkers, SP, NK1R and PKM2. This is consistent with previous findings, where human breast tumor cells were found to overexpress SP and NK1R [14, 20, 21], in addition to high expression of PKM2 [36, 37].

Standardization of immunohistochemical scoring of protein markers in cancer has not yet been accomplished. Additionally, different approaches were used to determine the optimal cut-off value of SP, NK1R, Ki-67 and PKM2 for predicting survival. In one study by Mehboob, et al., (2021) used a different immunohistochemistry scoring approach and found that SP was positive in more than two third (68%) of the investigated cases (23/34) and it was associated with tumor grade, indicating a negative prognostic value of SP in breast cancer [21]. Additionally, higher cytoplasmic expression of NK1R (PS > 10%) was associated with various prognostic factors such as TNM stage, grade, HR, and HER2 status [20]. Regarding PKM2, A relevant study by Huang, et al. (2018), high PKM2 (TS > 3) predicted poor survival in patients with urothelial carcinoma of the bladder [26]. Other studies that used different approaches and cut-off value definitions also found a significant impact of PKM2 expression on overall survival [28, 38]. The prognostic cut-off values for Ki-67 ranged from 10 to 25% [39–43], thus complicating the comparison of findings, but overall high expression of Ki-67 was associated with worse prognosis.

Through activation of NK1R, the SP/NK1R system regulates the proliferation, migration, and metastasis of breast cancer cells [44]. In our study, a statistically significant association was found between SP expression and pN stage, TNM, pT stage, axillary lymph node metastasis, and NK1R expression in HR-ve cases. These results support previous ones reporting the role of SP in promoting cancer metastasis [19, 45, 46]. Subsequently, through binding to NK1R, SP may also cause inflammation by inducing the production of reactive oxygen species (ROS) [47], and significantly reducing the expression of catalase and superoxide dismutase [48]. Furthermore, our results in comparing HR + ve/-ve cases, it is indicated that the proinflammatory SP and its receptor has a role in breast cancer by their interaction with hormone receptors. For instance, estrogens stimulate SP and contribute to sensitization of pain though proinflammatory α-adrenergic pathways, thus increasing neurogenic inflammation [49].

It is evident in the more aggressive HR + ve tumors, inflammation is a key factor in its development through the NF-κB pathway [50, 51]. This is further supported in our study, were we found SP expression was greatly associated with PKM2 expression and HER2 status in HR + ve tumors. PKM2 is highly expressed in tumor cells and has been shown to promote late pro-inflammatory cytokines though its interaction with hypoxiainducible factor 1α (HIF-1α) [52], and has a role as a critical mediator in inflammatory microenvironment of cancer [53, 54]. For instance, through activation of NF-κB signaling pathway, PKM2 is critical for the production of TNF-α and IL-1β in colorectal cancer [55]. Specially in breast cancer, PKM2 promotes tumor growth through regulating β-catenin and activating the wnt/β-catenin pathway [37]; thus, directly playing a critical role in the production of inflammatory cytokines and regulating the cell proliferation and tumorigenesis. Through promoting inflammatory pathways, PKM2 stimulates the phosphorylation of STAT3 to indorse the production of inflammatory cytokines [56, 57]. Moreover, regulating PKM2 expression through metabolic reprogramming gives cancer cells advantages to control of intracellular reactive oxygen species (ROS) concentrations and is critical for cancer cell survival to withstand oxidative stress.

Our survival analysis demonstrated a clear negative impact of SP expression level on overall survival of patients with HR-ve cases with low NK1R expression level. Since the proliferation biomarker Ki-67 is considered a prognostic factor for breast cancer, as such it is evident that SP expression is also significant in HR-ve cases with low Ki-67 index impacting overall survival. This result supports the potential role of SP/NK1R system as a therapeutic target in breast cancer.

The best approach to fighting cancer is an early diagnosis. For instance, a rather common diagnosis among women undergoing screening mammography is the evaluation of ductal carcinoma in situ (DCIS) [58]. In our study a trending, but not reaching significance, impact (p = 0.059) on overall survival in ER- cases with a DCIS history was found associated with high PKM2 expression. Similar risk factors for DCIS and invasive breast cancer point to a shared etiology for both diseases [58]. However, there is still debate regarding the precise ratio that can develop to invasive breast cancer and the rate of advancement. Consequently, progression biomarkers to allow monitoring of DCIS patients and determine the potentiality of DCIS to progress into invasive cancer are required.

Additionally, the overall survival in HR-ve cases was also negatively impacted by having high expression of PKM2. In breast cancer, targeting PKM2 might be a possible treatment. As such, the use of shikonin, a PKM2 inhibitor, prolonged animal survival and reduced tumor size as well as enhanced the sensitivity of human breast cancer cells to chemotherapy by paclitaxel [59]. However, considering the forked function of PKM2, downregulating or silencing PKM2 could possibly cause a wide range of response effects. Consequently, a systematic evaluation of PKM2’s therapeutic value is necessary.

Due to their aggressive clinical behavior and lack of known molecular targets for treatment, triple negative breast cancer (TNBC) is often associated with worse prognosis compared to those with other breast cancer subtypes. Several new research provided an insight of the role of SP/NK1R in TNBC. For instance, SP plays an unfavorable role in doxorubicin-associated killing of cardiomyocytes and induction of chemoresistance in TNBC. Thus, SP antagonism enhanced chemotherapy’s ability to kill resistant TNBC cells [60]. Further investigation on cisplatin use in combination with SP receptor (NK1R) antagonism showed to serve as a novel, more efficacious and safer therapeutic option than existing therapies for TNBC, as the levels of NK1R were significantly elevated in response to cisplatin in a rat neuronal cell line and in two TNBC cell lines [61]. The cell proliferation biomarker Ki-67 has been shown to be a valuable prognostic and predictive marker in triple-negative breast cancer [62–64]. Finally, most recently studies on PKM2 propose that targeting PKM2 inhibitor and its phosphorylation reverses the aggressive cancer phenotypes and sensitizes the TNBC cells [65, 66]. As such, PKM2 inhibitors could be an effective treatment for TNBC. Consequently, we recommend prospective large-scale future studies to evaluate the prognostic and predictive significance of PKM2 in breast cancer in combination with SP especially in TNBC.

Our study had various limitations. Firstly, small sample size thus limiting the generalizability of the data. Also, we advise in future studies for larger, multi-centered investigations that assess in terms of tumor recurrence and disease-free survival because the trial was single-centered and lacked data on recurrence status.

## Conclusion

As a conclusion, our results show that within HR-ve patients, higher expression level of SP is associated with pN3, positive axillary lymph node metastasis and high NK1R expression. Additionally, these cases had an overall worsening survival rate with high SP expression, low NK1R expression and low ki-67 levels. Moreover, high PKM2 with low SP had a negative impact on overall survival in HR-ve cases. These distinctive characteristics provide credence to the idea that HR-ve tumors are a morphologically and phenotypically distinct entity, and they also offer a justification for the investigation and application of more recent, promising agents in the management and treatment of HR-ve breast cancer. Future prospective studies with larger sample size are recommended to further evaluate the prognostic value of PKM2 and the SP/NK1R system in breast cancer.

## Data Availability

The datasets used and/or analysed during the current study are available from the corresponding author on reasonable request.
